# Time-Dependent Loss of miR-548c-3p and Activation of E2F3/FOXM1 in Breast Cancer: In Vitro and TCGA-Based Evidence for a Post-Transcriptional Mechanism

**DOI:** 10.3390/ijms27021052

**Published:** 2026-01-21

**Authors:** Buket Bozkurt, Durmus Ayan, Seyyid Mehmet Bulut

**Affiliations:** Department of Medical Biochemistry, Faculty of Medicine, Niğde Ömer Halisdemir University, Niğde 51240, Türkiye

**Keywords:** breast cancer, miR-548c-3p, E2F3, FOXM1, biomarkers

## Abstract

MicroRNAs are key post-transcriptional regulators in breast cancer, but their time-dependent dynamics and downstream oncogenic effects are not fully understood. miR-548c-3p has been proposed as a tumor suppressor, yet its temporal behavior and impact on cell cycle drivers remain unclear. This study investigated the time-dependent expression of miR-548c-3p and its post-transcriptional regulation of E2F3 and FOXM1 in MCF-7 breast cancer cells. Cells were analyzed at multiple time points (2–72 h) by quantitative real-time PCR to assess dynamic changes in miR-548c-3p, E2F3, and FOXM1 mRNA levels. Bioinformatic validation using TCGA-BRCA datasets and public platforms evaluated gene expression, promoter methylation, and prognostic significance. miR-548c-3p showed a progressive time-dependent decline, with the lowest levels at 72 h, whereas E2F3 and FOXM1 were significantly upregulated over time, supporting a post-transcriptional derepression mechanism. TCGA-based analyses confirmed overexpression and hypomethylation of E2F3 and FOXM1 in breast cancer, particularly in triple-negative tumors, and high expression of both genes was associated with poor survival. These findings indicate that time-dependent loss of miR-548c-3p contributes to E2F3 and FOXM1 activation through a post-transcriptional regulatory mechanism, highlighting this miRNA–oncogene axis as a potential prognostic signature and therapeutic target in breast cancer.

## 1. Introduction

Despite advancements in diagnostic methods, prognostic tools, and therapeutic strategies, the global incidence of breast cancer remains high, with more than one million new diagnoses recorded each year [1,2]. Breast cancer results from a combination of genetic alterations that lead to genomic instability and uncontrolled proliferation of mammary gland epithelial cells [3]. This heterogeneous cancer has four molecular subtypes: Luminal A (estrogen receptor [ER] and progesterone receptor [PR]), Luminal B (positive for ER, PR, and human epidermal growth factor 2-receptor [HER2]), HER2 (positive for HER2 only), and basal-like (negative for ER, PR, and HER2) [4,5]. While these subtypes offer a foundation for the treatment strategy required for a specific type of breast cancer, more precise treatments are required to optimize the therapy. The signs and symptoms of breast cancer may not be evident at an early stage. If an initial diagnosis and treatment are administered at an early stage of breast cancer progression, it is likely that the primary tumor cells have not metastasized, thus. Resulting in improved treatment outcomes. The molecular landscape of breast cancer is highly heterogeneous, encompassing diverse subtypes characterized by distinct genomic, transcriptomic, and proteomic profiles that significantly influence disease progression and treatment response. Advances in multi-omics technologies have revolutionized the capacity to identify prognostic biomarkers, uncover molecular networks, and enable precise molecular subtyping essential for targeted therapies [6]. Tumor microenvironment (TME) complexity, including the dynamic interactions between tumor cells, immune infiltrates, and stromal components, plays a pivotal role in modulating breast cancer progression and therapeutic outcomes. Particularly, immunosuppressive microenvironments characterized by elevated regulatory T cells and altered immune subsets contribute to tumor immune evasion and resistance to standard therapies [7,8]. Furthermore, recent systematic investigations have underscored the importance of microenvironmental factors, including the microbiome, inflammation, and cellular crosstalk, in shaping tumor behavior and patient prognosis [9,10]. These insights have catalyzed the development of sophisticated therapeutic strategies such as immune checkpoint inhibitors, antibody-drug conjugates, and precision-targeted agents that engage specific signaling pathways and immune components within the TME [11,12]. Building upon this evolving framework, this study integrated multi-omics data to elucidate novel biomarker candidates and explore their mechanistic involvement within breast cancer molecular networks and the intricate tumor microenvironment landscape, thereby contributing to the advancement of precision medicine approaches. Recent studies have indicated the involvement of several miRNAs in the genesis, development, metastasis, and medication resistance of breast cancer [13,14]. In particular, microRNAs (miRNAs) have shown potential for early diagnosis, prognosis, and as treatment plan biomarkers because of their regulatory role in breast cancer. miRNAs are unique biomolecules consisting of 18–25 nucleotides of non-coding RNA that can regulate gene expression of their target messenger RNA via post-transcriptional modification [15] in the genesis, development, metastasis, and medication resistance of breast cancer. In particular, because of their regulatory role in breast cancer, miRNAs have shown potential as biomarkers in the disease’s early diagnosis, prognosis, and treatment planning. Moreover, miRNAs have been demonstrated to play pivotal regulatory roles in breast cancer, functioning as both oncogenes and tumor suppressors [16]. According to a comparative analysis of multiple cell types, numerous miRNAs can serve as biomarkers for breast cancer treatment. E2Fs, transcription factors that play a critical role in cell proliferation, are also involved in the repair of double-strand breaks in DNA damage by members such as E2F transcription factor 1 (E2F1) [17,18]. However, by modulating BRCA1 transcription in breast cancer, E2F1 promotes homologous recombination repair and radioresistance [19]. In an in silico analysis conducted to explore the influence of E2Fs on breast cancer treatment resistance, E2F3 was found to contribute to S phase entry, DNA damage repair, DNA replication processes, and resistance to radiotherapy and chemotherapy. Forkhead box M1 (FOXM1) is a downstream E2F3 signaling molecule that mediates E2F3’s effects on breast cancer cells. A study of the miR-548 gene family enrichment pathway revealed that 69 human miR548 genes, which comprise the broader, poorly conserved primate-specific miRNA gene family, are found on nearly every human chromosome. These genes have been implicated in several human disorders [20]. According to some studies, miR-548c-3p regulates a wide range of cancers [21]. Including osteosarcoma [22], glioma [23], gastric cancer [24], breast cancer [21,25], prostate cancer [26], esophageal cancer [27], thyroid cancer [28], and osteosarcoma [22]. However, the degree of its expression and its underlying mechanism in breast cancer remain unclear. These comparative studies on multiple breast cancer cells isolated RNA at only one specific time point. This can be misleading because gene expression is a dynamic process, and cells can display a transcriptional pulse of rapid gene expression at different times. Since modulating the miRNA expression is a powerful means of controlling the target gene expression during both physiological and pathological transitions in organisms, time-dependent gene expression analysis is becoming increasingly important for researchers to understand cancer cell behavior [29].

In this study, we address a critical research gap by investigating the time-dependent dynamics of breast cancer-related miRNAs instead of relying on single time point analyses. A high-throughput screening approach was followed to profile the expression of 84 breast cancer–associated miRNAs in ER-positive MCF-7 cells across six consecutive time points (2 h, 8 h, 16 h, 24 h, 48 h, and 72 h). Among these, miR-548c-3p emerged as the most prominently and progressively downregulated miRNA. We then integrated in vitro time-resolved expression analyses with large-scale TCGA-based bioinformatic validation to explore the post-transcriptional relationship between miR-548c-3p and its key oncogenic targets, E2F3 and FOXM1. By combining temporal miRNA kinetics with transcriptional, epigenetic, and survival data, this study posits mechanistic insights specific to MCF-7 cells, while TCGA analysis offers broader clinical context, and offers, through TCGA analysis, a broader clinical context.

## 2. Results

### 2.1. The miRNA Expression Profiles of MCF-7 Cell Lines at Different Time Points

**Figure 1:**
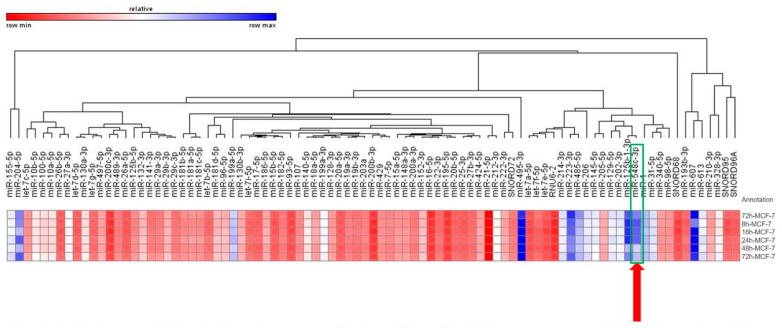
The following report details the alterations in the expression profile of microRNAs associated with breast cancer, as illustrated by a heat map. While a significant change in the expression of three microRNAs was detected at 8 h, a significant change in the expression of twenty-six microRNAs was determined in MCF-7 cells at 16 h. Four miRNAs changed significantly in MCF-7 at 24 h. Five miRNAs were found at 48 h. Six miRNAs were significantly determined at 72 h. The expression scale is shown under each heat map. Blue boxes represent lowexpression, while red boxes represent high expression.

The expression of 84 breast cancer-related miRNAs was analyzed in MCF-7 cells at six different time periods ranging from 2 h to 72 h. The statistical analysis revealed that three miRs, hsa-miR-18a-5p (−2.0166; *p* < 0.0001), hsa-miR-7-5p (−2.1263; *p* < 0.001), and hsa-miR-199a-5p (−2.1064; *p* < 0.044), were differentially expressed by at least two-fold at 8 h (Figure 1). Additionally, 26 miRs, hsa-miR-140-5p (−2.2683; *p* < 0.0005), hsa-miR-18a-5p (−2.6988; *p* < 0.00003), hsa-miR-19a-3p (−2.3267; *p* < 0.00009), hsa-miR-19b-3p (−2.312; *p* < 0.00003), hsa-miR-20b-5p (−2.0214; *p* < 0.000004), hsa-miR-21-5p (−2.0563; *p* < 0.0008), hsa-miR-22-3p (−2.0802; *p* < 0.0003), hsa-miR-27b-3p (−2.1164; *p* < 0.000003), hsa-miR-29a-3p (−2.2273; *p* < 0.0001), hsa-miR-29b-3p (−2.7499; *p* < 0.000008), hsa-miR-29c-3p (−2.2051; *p* < 0.00014), hsa-miR-424-5p (−2.9691; *p* < 0.00008), hsa-miR-429 (−2.088; *p* < 0.0000007), hsa-miR-7-5p (−2.0257; *p* < 0.0001), hsa-miR-96-5p (−3.3386; *p* < 0.0001), hsa-miR-141-3p (−2.4255; *p* < 0.000009), hsa-miR-148a-3p (−2.2903; *p* < 0.0002), hsa-miR-152-3p (−2.0381; *p* < 0.0006), hsa-miR-15a-5p (−2.1542; *p* < 0.00001), hsa-miR-16-5p (−2.4966; *p* < 0.001), hsa-miR-17-5p (−2.199; *p* < 0.001), hsa-miR-199a-5p (−2.1674; *p* < 0.002), hsa-miR-195-5p (−2.1842; *p* < 0.0001), hsa-miR-200a-3p (−2.3433; *p* < 0.00001), hsa-miR-186-5p (−2.2683; *p* < 0.00002) and hsa-miR-204-5p (6.8711; *p* < 0.05) demonstrated varied expression levels at 16h in MCF-7 (over two-fold up-/downregulated) (Figure 1). While the fourth stage (24 h) had four miRNAs that were over two-fold upregulated, hsa-miR-100-5p (2.0112; *p* < 0.004), hsa-miR-485-5p (9.7816; *p* < 0.001), let-7f-5p (2.4085; *p* < 0.001), and hsa-miR-214-3p (2.2777; *p* < 0.003), there was one miRNA, hsa-miR-199a-5p (−2.0691; *p* < 0.002), that was downregulated by over two-fold (Figure 1). While the expression of hsa-miR-485-5p (−9.1982; *p* < 0.002) and hsa-miR-548c-3p (−22.2458; *p* < 0.007) decreased at 48 h, that of let-7f-5p (2.1502; *p* < 0.001), hsa-miR-145-5p (2.3723; *p* < 0.0009), and hsa-miR-202-3p (4.0369; *p* < 0.001) significantly increased (Figure 1). Compared with expression at 2 h, there were four miRNAs (hsa-miR-145-5p (3.8476; *p* < 0.01), hsa-miR-202-3p (2.6273; *p* < 0.03), hsa-miR-205-5p (2.3936; *p* < 0.001), and hsa-miR-548c-3p) that were differentially expressed in MCF-7 cells, including two miRNAs (hsa-miR-141-3p (−2.1475; *p* < 0.00001) and hsa-miR-328-3p (−2.0824; *p* < 0.00003)) that were downregulated at 24 h (Figure 1). Additionally, miR-548c-3p was dramatically under-expressed at 48 h (fold change = −22.24, *p* = 0.0073) and 72 h (fold change = −16.24, *p* = 0.0154) compared to the expression level at 2 h isolation in MCF cells, while miR-548c-3p was significantly under-expressed at 16 h (fold change = −4.29, *p* = 0.0122) and at 24 h (fold change = −2.38, *p* = 0.0118).

### 2.2. Results of E2F3 and FOXM1 mRNA Expressions

**Figure 2:**
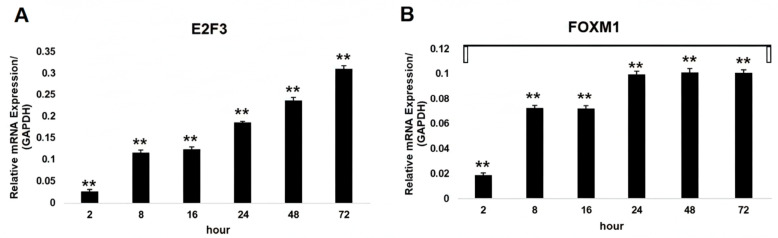
E2F3 and FOXM1mRNA expression in MCF-7s. The expression of target genes in MCF-7s from each group was detected by quantitative RT-PCR (target genes were normalized to the housekeeping gene GAPDH). (**A**) E2F3 mRNA expression at 2 h, 8 h, 16 h, 24 h, 48 h, and 72 h; (**B**) FOXM1 mRNA expression at 2 h, 8 h, 16 h, 24 h, 48 h, and 72 h; ** *p* < 0.001 vs. control.

As Figure 2 shows, the expression levels of E2F3 in MCF-7 cells significantly changed in a time-dependent manner at different time points (*p* < 0.01). The E2F3 mRNA expression level significantly increased at 8 h, 16 h, 24 h, 48 h, and 72 h compared with the expression level at 2 h in a time-dependent manner (*p* < 0.01) (Figure 2A). The highest increase was observed at 48 h and 72 h (*p* < 0.01) (Figure 2A). Additionally, the FOXM1 mRNA levels were significantly upregulated compared with those at 2 h at 8 h, 16 h, 24 h, 48 h, and 72 h (*p* < 0.01) (Figure 2B).

### 2.3. Possible Pathway Analysis of Differentially Expressed miRNAs

A pathway analysis of the putative target genes of miR-548c-3p performed using the DIANA TOOLS mirPath program revealed that these genes play key roles in various pathways, such as the pathways in cancer (hsa05200; *p* value: 6.547202 × 10^−19^; #genes:118; #miRNAs:1), transcriptional mysregulationin cancer (hsa05202; *p* value: 1.784339 × 10^−17^; #genes:71; #miRNAs:1), Wnt signaling pathway (hsa04310; *p* value: 4.427122 × 10^−7^; #genes:49; #miRNAs:1), mTOR signaling pathway (hsa 04150; *p* value: 0.000005919155; #genes:23; #miRNAs:1), and p53 signaling pathway (hsa04115; *p* value: 1.094354 × 10^−9^; #genes:27; #miRNAs:1).

### 2.4. Bioinformatic Analysis Results

#### 2.4.1. Expression and Promoter Region Methylation Results of E2F3 and FOXM1

**Figure 3:**
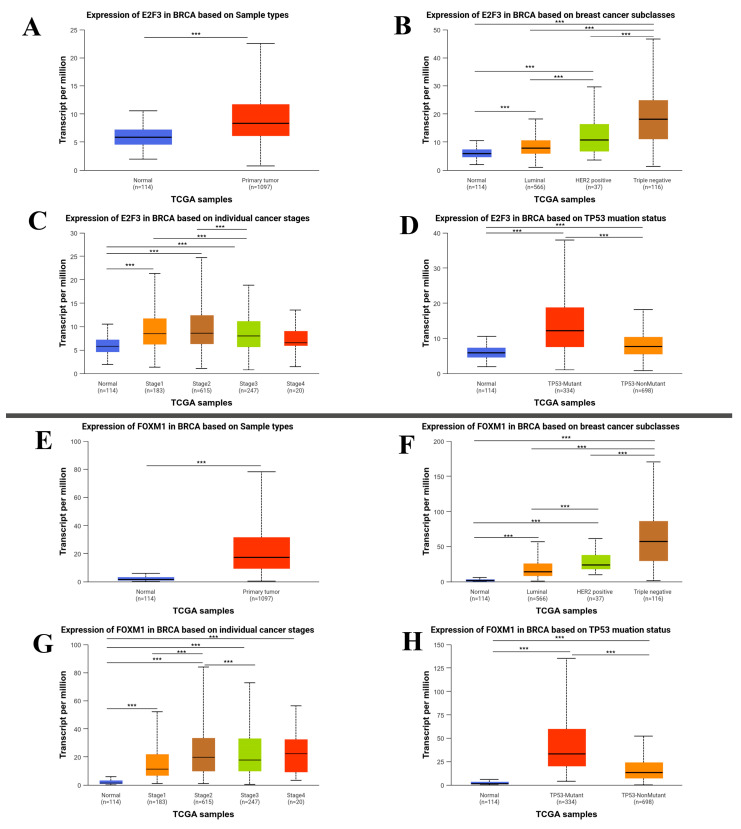
This figure illustrates the expression patterns of E2F3 and FOXM1 in breast cancer (BRCA) based on TCGA data. Panels (**A**–**D**) show E2F3 expression according to sample type (normal breast tissue vs. primary tumor), molecular subtypes (Luminal, HER2-positive, and triple-negative), individual tumor stages, and TP53 mutation status (TP53-mutant vs. TP53-nonmutant), respectively. Panels (**E**–**H**) present FOXM1 expression using the same stratifications. Gene expression levels are reported as transcripts per million (TPM). Box plots display the median and interquartile range, with whiskers indicating minimum and maximum values. Statistical significance is denoted as follows: (***) *p* < 0.001.

**Figure 4:**
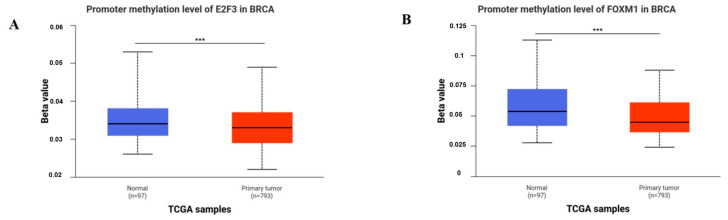
Promotor methylation level of E2F3 in BRCA (**A**); promotor methylation level of FOXM1 in BRCA (**B**). (***) *p* < 0.001.

E2F3 and FOXM1 were statistically upregulated in the BRCA cohort compared with normal adjacent tissues (*p* = 1.62 × 10^−12^, respectively) (Figure 3A–E). Among breast cancer subtypes, the highest expression increase in E2F3 and FOXM1 was observed in TNBC based on TCGA data; however, these bioinformatic findings do not imply that our in vitro results can be generalized to TNBC (*p* < 1 × 10^−12^, *p* = 1.62 × 10^−12^, respectively). FOXM1 expression was statistically upregulated in TNBC in the TCGA dataset; however, this reflects clinical data and is different from our MCF-7 in vitro model (*p* = 1.62 × 10^−12^, *p* < 1 × 10^−12^, *p* = 1.62 × 10-12, *p* = 2.22 × 10^−5^, respectively) (Figure 3B–F). On the other hand, E2F3 expression was upregulated in stage I, stage II, and stage III compared with normal tissue (*p* = 3.67 × 10^−11^, *p* = 1.62 × 10^−12^, 1.70 × 10^−12^, respectively), while there was no statistical difference in stage IV compared with normal adjacent tissue (*p* = 0.128) (Figure 3C–G). On the other hand, in cases with TP53 mutation, both E2F3 expression [normal vs. TP53 mutant (*p* = 1.62 × 10^−12^), normal vs. TP53 nonmutant (*p* = 1.62 × 10^−12^), TP53 mutant vs. TP53 nonmutant (*p* = 1.11 × 10^−16^)], and FOXM1 expression [normal vs. TP53 mutant (*p* = 1.62 × 10^−12^), normal vs. TP53 nonmutant (*p* = 1.62 × 10^−12^), TP53 mutant vs. TP53 nonmutant (*p* = 1.62 × 10^−12^)] were found to be statistically upregulated compared to the normal adjacent tissue and the tumoral tissue without TP53 mutation (Figure 3D–H). E2F3 and FOXM1 were statistically hypomethylated in the BRCA cohort compared with normal adjacent tissues (*p* = 2.72 × 10^−3^, *p* = 3.71 × 10^−5^, respectively) (Figure 4).

#### 2.4.2. Survival Analysis Results of E2F3 and FOXM1

**Figure 5:**
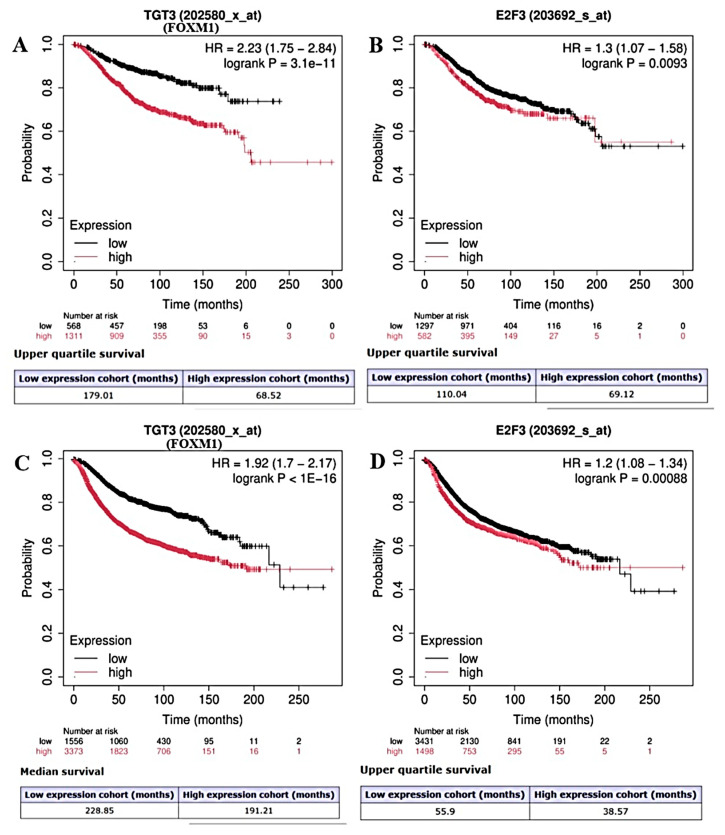
Kaplan–Meier survival analyses showing the association between gene expression levels of FOXM1 and E2F3 and patient outcomes in breast cancer. Panel (**A**) illustrates the relationship between overall survival (OS) and FOXM1 (probe ID: 202580_x_at), while panel (**B**) shows the association between OS and E2F3 (probe ID: 203692_s_at). Panel (**C**) presents the association between relapse-free survival (RFS) and FOXM1, and panel (**D**) shows the association between RFS and E2F3. Patients were stratified into high- and low-expression groups based on median gene expression levels. Survival probabilities are plotted over time (months). Hazard ratios (HRs) with 95% confidence intervals (CIs) and log-rank *p* values are indicated within each panel. Numbers at risk for each group are shown below the curves. High expression of both FOXM1 and E2F3 is associated with poorer OS and RFS compared with low expression.

The elevated expression levels of E2F3 and FOXM1 were statistically associated with shorter OS (*p* = 0.0093, *p* = 3.1 × 10^−11^, respectively) (Figure 5A,B) and shorter RFS (*p* < 1 × 10^−16^, *p* = 0.00088, respectively) (Figure 5C,D).

#### 2.4.3. MicroRNA Target Analysis Result

**Figure 6:**
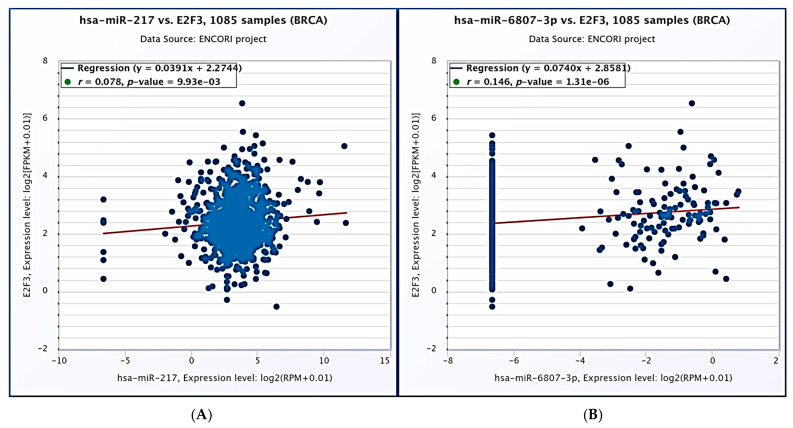
Correlation analysis between E2F3 expression and selected microRNAs in breast cancer (BRCA) samples based on the ENCORI database. (**A**) Correlation between hsa-miR-217 and E2F3 expression levels in 1085 BRCA samples. (**B**) Correlation between hsa-miR-6807-3p and E2F3 expression levels in 1085 BRCA samples. Expression levels are presented as log2(FPKM + 0.01) for E2F3 and log2(RPM + 0.01) for miRNAs. Linear regression lines are shown in red. Correlation coefficients (r) and *p* values are indicated in each panel.

**Figure 7:**
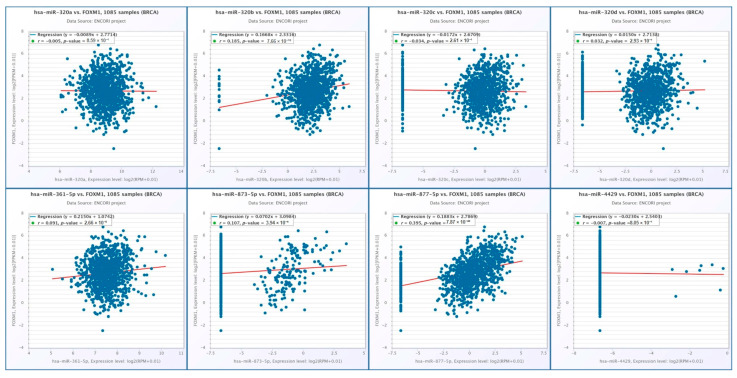
Correlation analysis between FOXM1 expression and selected microRNAs in breast cancer (BRCA) samples based on the ENCORI database. Correlation plots show the relationships between FOXM1 and the following miRNAs in 1085 BRCA samples: hsa-miR-320a, hsa-miR-320b, hsa-miR-320c, hsa-miR-320d, hsa-miR-361-5p, hsa-miR-873-5p, hsa-miR-877-5p, and hsa-miR-4429. Expression levels are presented as log2(FPKM + 0.01) for FOXM1 and log2(RPM + 0.01) for miRNAs. Red lines indicate linear regression. Correlation coefficients (r) and corresponding *p* values are shown within each panel.

While hsa-miR-217 and hsa-miR-6807-3p were associated with E2F3, conserved hsa-miR-877-5p, hsa-miR-873-5p, hsa-miR-361-5p, hsa-miR-320b, hsa-miR-320a, hsa-miR-4429, hsa-miR-320d, hsa-miR-320c and hsa-miR-325-3p were associated with FOXM1. According to the results obtained from the ENCORİ database, a significant positive correlation was found between the E2F3 expression and the expressions of hsa-miR-217 and hsa-miR-6807-3p (r = 0.079, *p* = 9.93 × 10^−3^; r = 0.146, *p* = 1.31 × 10^−6^, respectively) (Figure 6). In contrast, a significant positive correlation was found between the FOXM1 expression and the expressions of hsa-miR-361-5p, hsa-miR-320b, hsa-miR-873-5p, and hsa-miR-877-5p (r = 0. 091, *p* = 2.66 × 10^−3^, r = 0.185, *p* = 7.66 × 10^−10^, r = 0.107, *p* = 3.94 × 10^−4^, r = 0.395, *p* = 7.87 × 10^−42^, respectively). However, no negative or positive correlation was found with others (*p* > 0.05) (Figure 7).

#### 2.4.4. Gene-Gene Interaction Result

**Figure 8:**
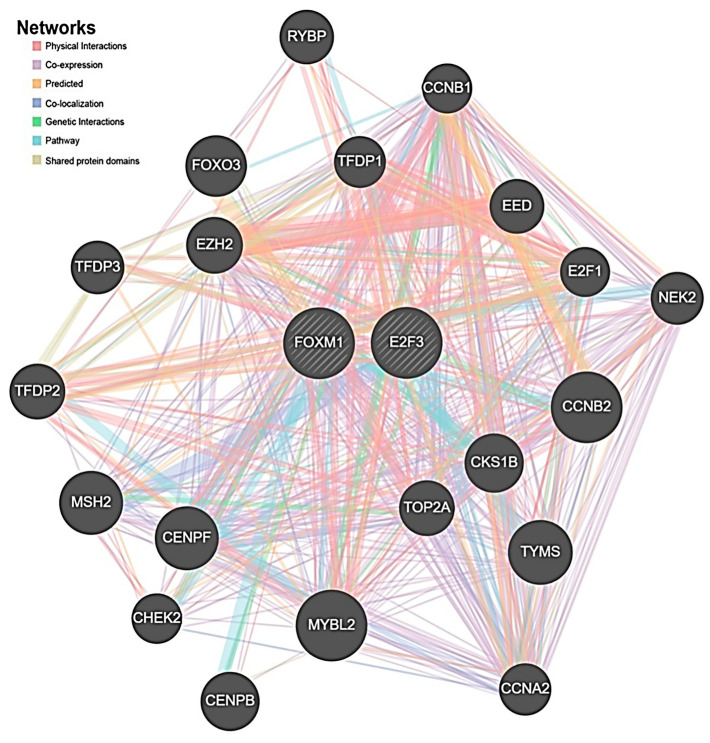
Gene–gene interaction network of E2F3, FOXM1, and their functionally related genes generated using the GeneMANIA web tool. The network illustrates known and predicted interactions based on multiple evidence types, including physical interactions, co-expression, predicted interactions, co-localization, genetic interactions, pathway associations, and shared protein domains. E2F3 and FOXM1 are highlighted as central hub genes within the network, showing strong connectivity with key regulators of cell cycle progression, DNA replication, DNA damage response, and mitotic control, including CCNB1, CCNB2, CCNA2, TFDP1, TFDP2, TFDP3, CKS1B, TOP2A, MYBL2, EZH2, CHEK2, MSH2, and CENPF.

Most of the physical interactions were observed (77.64%), and the co-expression ratio was found to be 8.01%; the predicted ratio was 5.37%. The colocalization ratio was 3.63%, the genetic interaction ratio was 2.87%, the pathway ratio was 1.88%, and the shared protein-domain ratio was 0.60% (Figure 8).

## 3. Discussion

Owing to the molecular complexity of breast cancer, a heterogeneous disease characterized by many different morphologies, genetic structures, and therapeutic options [30], proper therapeutic decisions are limited. In recent years, several studies have demonstrated that miRNAs are highly expressed in several cancers, including breast cancer, with the findings of these studies suggesting that miRNAs may be useful therapeutic biomarkers. Recent studies have also monitored transcriptional pulsing in eukaryotes at multiple time points [31], finding gene-on and gene-off times to be highly important for post-transcriptional regulation [29]. However, most of the studies selected only a single time point for RNA isolation. In this study, the expression of 84 miRNAs associated with breast cancer in ER-positive (+) minimally invasive luminal epithelial MCF-7 breast cancer cells was analyzed at multiple time points (2 h, 8 h, 16 h, 24 h, 48 h, and 72 h). A significant decrease in the expression of miR-548c-3p was found at different time points. The subsequent analysis sought to ascertain the effect of miR-548c-3p on the transcription of E2F3 and FOXM1 genes at the same time points by conducting in vitro experiments using MCF-7 cells and bioinformatic analyses to determine its possible effects on breast cancer. The miR-548 family of microRNAs, comprising 69 human members, is present in almost all human chromosomes and plays an important role in various diseases [20]. Mature miR-548c-3p is derived from the precursor molecule miR-548c, which belongs to the miR-548 family of miRNAs. A comprehensive literature review indicated that miRNA-548c-3p has a regulatory function in numerous types of cancers, including prostate cancer, esophageal cancer, thyroid carcinoma, gastric cancer, and breast cancer [21]. The miRNA-548c-3p expression was found to be reduced in a time-dependent manner compared to the expression levels observed at 2 h. However, the most significant decrease was observed at 48h and 72 h of the experimental period, with decreases of 22- and 16-fold, respectively. Guo et al. (2019) found decreased expression of miR-548c-3p in MCF-7 cells and showed that this decrease was associated with the upregulation of HIF1A-AS2 and HIF-1α, which play important roles in cancer progression [21]. In the present study, we found a statistically significant decrease in the expression level of miR-548c-3p from 16 h to 72 h in comparison with the level observed in t 2 h. Notably, the most significant decrease was observed at 48 h, with a 22-fold decrease in response. A further study of the relevant literature revealed that the expression levels of microRNA 548 were lower in breast cancer cells than in normal healthy control cells. Furthermore, the same study demonstrated that the upregulation of miR-548 may improve breast cancer progression [32]. In particular, the regulation of miRNA-548c-3p and its interaction with HIF1A-AS2 and HIF-1α in MCF-7 cells suggests that it may have potential as a biomarker and therapeutic target in breast cancer treatment. Evidently, miR-548c-3p substantially influences the regulatory processes governing the behavior of breast cancer cells, particularly MCF-7 cells. miRNAs function by impeding the various hallmarks of cancer, including uncontrolled cell proliferation, malignant invasion, and epithelial–mesenchymal transition (EMT), while concurrently inducing a state of cellular senescence [33]. We found that the post-transcriptional regulation of MCF-7 cells exhibits time-dependent variability. Additionally, the E2F3 mRNA expression, which is considered a breast cancer promoter, was found to be increased at 8 h, 16 h, 24 h, 48 h, and 72 h compared with the expression at 2 h in inverse proportion to the miR-548c-3p expression. Wei et al. found that breast cancer tissues exhibited elevated E2F3 mRNA levels compared with normal breast tissues, particularly in breast cancer cell lines, including MCF-7. This has been shown to induce the formation of breast cancer stem cells as well as chemoresistance and radioresistance. Furthermore, the expression level of E2F3 was found to be higher in breast cancer cell lines, including MCF-7 cells [34]. In the context of in vivo breast cancer model studies, the deletion of E2F3 has been found to have a pivotal function in tumorigenesis, primarily by virtue of its capacity to retard the onset of tumor formation [35,36,37]. The results demonstrated that FOXM1 expression levels were elevated at all designated time periods. While FOXM1 is responsible for cell cycle transition in normal cells, it also plays an active role in cell proliferation, DNA replication, and DNA damage repair processes in breast cancer cells [38,39]. Wei et al. found that FOXM1 overexpression restored E2F3-associated phenotypes [34]. Moreover, the inhibition of FOXM1 has been found to attenuate breast cancer cell growth in vitro and in vivo. FOXM1, a transcription factor, induces chemoresistance in breast cancer cells through a mechanism that enables the accurate and reliable repair of DNA double-strand breaks. These pathways revealed miR-miR-548c-3p to be principally involved in cancer and different neoplasms, although the relevance of this information was relative, as these miRNA target genes remained to be validated. Accordingly, we used the DIANA miRPath 2.0 software to analyze the main potentially targeted genes of miR-548c-3p. The functional pathway analysis indicated that these miRNAs play key roles in the p53 and VEGF signaling pathways. miRNAs can act as oncogenes or tumor suppressors to inhibit or aggravate the expression of cancer-related target genes [40,41]. Moreover, the bioinformatic analysis results indicated that E2F3 and FOXM1 are not epigenetically regulated, thus suggesting their role as significant oncogenes in breast cancer. Notably, the TCGA datasets revealed the elevated expression of these genes in TNBC; however, this bioinformatic association does not imply that our MCF-7 in vitro findings are directly generalizable to TNBC biology. These TNBC-related observations derived from TCGA analyses were incorporated solely to provide clinical and molecular context for the in vitro findings and were not intended to imply direct mechanistic conservation between MCF-7 cells and TNBC tumors. The temporal regulatory dynamics observed in the MCF-7 cells represent an ER-positive model system, and thus cannot be directly extrapolated to triple-negative breast cancer biology. The recent advances in multi-omics profiling have revealed that tumor progression is shaped not only by intrinsic transcriptional programs but also by the surrounding immune microenvironment, which integrates stromal, immune, extracellular matrix, and vesicle-mediated signaling networks. Multi-layered immune microenvironment analyses, including the characterization of tumor-associated macrophage (TAM) polarization states, dendritic cell dysfunction, NK-cell cytotoxic imbalance, and cytokine-driven inflammatory loops, highlight that immune remodeling is a central determinant of cancer aggressiveness and therapeutic resistance [42]. As shown in the comprehensive immune-microenvironment review [42], coordinated alterations in immune cells, chronic inflammation, and extracellular matrix (ECM) remodeling contribute to transcriptional heterogeneity—thereby mechanistically aligning with the temporal derepression patterns of E2F3 and FOXM1 in our model. Moreover, emerging evidence indicates that the microbiome modulates tumor-associated immune pathways, epigenetic states, and oncogenic transcription factor networks, adding an additional regulatory layer to tumor biology [42,43]. Within this integrated biological framework, the time-dependent miR-548c-3p/E2F3/FOXM1 axis we identified may represent one component of a broader regulatory landscape in which transcription factor activation, immune-microenvironment interactions, and microbiome-associated signals converge to drive aggressive breast cancer phenotypes. Our findings thus complement multi-omics precision oncology approaches by introducing a dynamic temporal regulatory dimension. Furthermore, genetic alterations in TP53, a tumor suppressor, have been observed to result in increased expressions of E2F3 and FOXM1. The loss of these tumor suppressor mechanisms has been shown to trigger the uncontrolled progression of the cell cycle and failure to repair DNA damage. Given the direct interaction between E2F3 and the FOXM1 and TP53 pathways, elevated FOXM1 levels in the presence of TP53 mutations may contribute to an increase in cancer aggressiveness. Our gene–gene interaction results suggest that E2F3 and FOXM1 show direct physical interactions and that these two genes work together or play important roles in the same signaling pathway, but can also be activated by independent mechanisms. The bioinformatics analysis results demonstrated that both E2F3 and FOXM1 can be regarded as diagnostic and prognostic biomarkers. Elevated E2F3 and FOXM1 expressions have been correlated with a more aggressive breast cancer phenotype and a poorer patient prognosis. Furthermore, FOXM1 has been shown to have a more pronounced prognostic effect than E2F3.

In conclusion, our study provides comprehensive insights into the post-transcriptional regulation of miR-548c-3p and its impact on the E2F3 and FOXM1 expression in MCF-7 breast cancer cells over multiple time points. We found the level of miR-548c-3p decreases significantly over time, particularly at 48 h and 72 h, which is in turn associated with the upregulation of E2F3 and FOXM1. The inverse relationship between miR-548c-3p and these oncogenes suggests their potential regulatory role in breast cancer progression.

### Limitations

This study has several limitations. First, since we performed the experimental analyses using a single estrogen receptor–positive breast cancer cell line (MCF-7), our findings may not fully reflect the molecular dynamics of other breast cancer subtypes, particularly triple-negative breast cancer (TNBC), despite the supporting TCGA-based bioinformatic validation. Second, although the inverse relationship between miR-548c-3p and its predicted oncogenic targets E2F3 and FOXM1 was strongly supported by our time-dependent expression analyses and large-scale public datasets, we did not include direct functional validation experiments such as Western blotting, dual-luciferase reporter assays, or gain-/loss-of-function approaches using miRNA mimics or inhibitors. These experiments will be critical in future studies to confirm direct miRNA–target interactions and establish mechanistic causality. Third, while the bioinformatic analyses provided robust prognostic and epigenetic correlations, the in vivo validation using animal models was beyond the scope of this study. Finally, although dynamic expression profiling was conducted across multiple time points, additional high-resolution temporal analyses may further refine the precise kinetics of the miR-548c-3p–E2F3–FOXM1 regulatory axis. Future studies addressing these limitations will be essential to fully clarify the mechanistic and translational potential of this regulatory network in breast cancer.

## 4. Materials and Methods

### 4.1. Cell Culture

The human breast adenocarcinoma cell line MCF-7 was kindly provided by Prof. Dr. Bahadır Öztürk’s Laboratory, Department of Medical Biochemistry, Faculty of Medicine, Selçuk University (Konya, Türkiye), and transferred to our laboratory in 2021. Upon receipt, the cells were expanded and cryopreserved in liquid nitrogen until use. Cell line identity was verified based on morphological characteristics and growth behavior consistent with standard MCF-7 features. Before initiating the experiments, mycoplasma contamination was tested using the EZ-PCR Mycoplasma Detection Kit (Sartorius, Göttingen, Germany) according to the manufacturer’s instructions and was confirmed to be negative.

The human breast cancer-derived cell line (MCF-7) was cultured in a humidifier containing 5% CO_2_ at 37 °C. The MCF-7 cells were cultured in Dulbecco’s Modified Eagle medium (DMEM) supplemented with 1 g/L of D-glucose, 4 mM L-glutamine, 1 mM pyruvate (GIBCO, Thermo Fisher Scientific, Waltham, MA, USA), 10% fetal bovine serum (FBS) (GIBCO, Thermo Fisher Scientific, Waltham, MA, USA), and 1% penicillin-streptomycin (GIBCO). This study did not involve human participants or experimental animals. All experiments were performed using a commercially available human breast cancer cell line (MCF-7). Therefore, ethical approval and informed consent are not applicable.

### 4.2. RNA Isolation

Following the manufacturer’s instructions, total RNAs, including miRNA, were extracted from the MCF-7 cells at 2 h, 8 h, 16 h, 24 h, 48 h, and 72 h using the High Pure miRNA Isolation Kit (Roche Molecular Biochemicals, Mannheim, Germany).

### 4.3. Complementary DNA (cDNA) Synthesis and Pre-Amplification

The purity and integrity of the extracted total RNA were evaluated using a spectrophotometer and an Agilent Bioanalyzer 2100 (Agilent RNA 6000 Nano, Santa Clara, CA, USA). Subsequently, the miScript II RT Kit cDNA synthesis kit (Qiagen, Hilden, Germany) was used to convert 2 µL of the total RNA samples to cDNA for the identification of miRNAs. For a total reaction volume of 7 µL, 2 µL of the total RNA was mixed with 5 µL of the reverse transcription reaction mix (10× miScript Nucleics Mix, 5× miScript HiSpec Buffer, miScript Reverse Transcriptase Mix, DNase, and RNase-free water). A Piko Thermal Cycler (Thermo Scientific, Waltham, MA, USA) was used for reverse transcription. The conditions for cDNA synthesis were as follows: 37 °C for 60 min, 95 °C for 5 min, and subsequently 4 °C. Subsequent to reverse transcription, pre-amplification was conducted using the TaqMan PreAmp Master Mix (Applied Biosystems; Thermo Fisher Scientific, Waltham, MA, USA) and Human Primer Pools Set (Exigon; Vedbæk, Denmark). The pre-amplification process entailed transferring 2 µL of the cDNA samples into a sterile 96-well plate, adding 8 µL of the pre-amplification suspension buffer (5× miScript PreAmp Buffer, HotStartTaq DNA Polymerase, Primer Tool, PreAMP Universal Primer) on top of the cDNAs, and subsequent pipetting up and down three or four times to ensure thorough mixing. The pre-amplification cycling conditions were 95 °C for 10 min, 94 °C for 30 s, and 60 °C for 3 min (12 cycles). Subsequently, primer dimers were eliminated from the pre-amplified cDNA samples by adding 1 µL of exonuclease solution (exonuclease solution and DNA suspension buffer). The exonuclease cycling conditions were 37 °C for 15 min and 95 °C for 5 min.

### 4.4. Multiplexed Polymerase Chain Reaction at High Throughput

This study utilized a high-throughput Bio-Mark real-time PCR system (Fluidigm, South San Francisco, CA, USA) to conduct high-throughput multiplexed polymerase chain reaction (h-PCR) studies on a BioMark 96.96 dynamic array chip.

In total, 84 primers for the human breast cancer miRNA PCR array, including miR-548c-3p, were procured using the miScript Primer Assay (Table 1). We focused on the evaluation of small nucleolar RNA, C/D box 68 (*SNORD68*), C/D box 72 (*SNORD72*), C/D box 95 (*SNORD95*), and C/D box. Moreover, 96A (*SNORD96A*), small nucleolar RNA, C/D box 61 (*SNORD61*), and RNA, U6 small nuclear 2 (*RNU6-2*), were evaluated as potential housekeeping genes, with *SNORD61* serving as an internal control. Subsequent to the standardization of all data to this internal control, fold regulation was calculated using relative quantification (RQ = 2^−ΔΔCT^). Moreover, the thermal procedure was performed using the Biomark System in the manner described below: 24 cycles of 94 °C for 15 s, 55 °C for 30 s, and 70 °C for 30 s, followed by 50 °C for 2 min, 70 °C for 30 min, 25 °C for 10 min, and 95 °C for 10 min. The process was concluded with a melting curve cycle at 60–95 °C for 1 min.

### 4.5. Pathway Analysis of Variably Expressed miRNAs

We used the DIANA-microT v5.0 algorithm via The DIANA TOOLS (http://diana.imis.athena-innovation.gr, accessed on 7 December 2025) platform was utilized to examine prospective targets and investigate the likely involvement of miR-548c-3p in breast cancer. We hypothesized that the selected miRNAs would target a large number of genes. In the subsequent stage of the research, the viable pathways that comprised the probable target gene of miR-548c-3p were examined using the mirPath 2.0 software from the DIANA TOOLS suite.

### 4.6. Real-Time PCR for the mRNA Expressions of E2F3 and FOXM1

The MCF-7 cells were seeded at a density of 5 × 10^4^ cells/cm^2^ in 60 mm cell culture dishes. When they reached confluency, DMEM and 10% FBS were added. After culturing the MCF-7 cells, total RNA was isolated from each cell using a monophasic solution of phenol and guanidine isothiocyanate after 2 h, 8 h, 16 h, 24 h, 48 h, and 72 h.

### 4.7. cDNA Synthesis and Quantitative Reverse Transcription-Polymerase Chain Reaction (RT-PCR)

Complementary DNA synthesis was performed according to the manufacturer’s instructions. The specific transcripts of *E2F3* and *FOXM1* (Table 2) were measured by quantitative real-time polymerase chain reaction (q-PCR) using the SYBR Green qPCR Master Mix and analyzed with BIORAD-CFX Connect.

### 4.8. Bioinformatics Analysis

#### 4.8.1. Expression and Promoter Region Methylation Analysis of E2F3 and FOXM1

UALCAN (https://ualcan.path.uab.edu/, accessed on 7 December 2025) is a comprehensive, user-friendly, and interactive web resource for analyzing cancer transcriptomic data. It aims to facilitate the exploration of The Cancer Genome Atlas (TCGA) data and help researchers analyze gene expression profiles and perform in-depth analyses of various cancer types. It offers insights into the methylation status of gene promoters, thereby allowing users to compare the methylation levels between the tumor and normal samples and compare the correlation between the expression levels of the two genes. Moreover, it is useful for identifying the potential gene interactions and pathways [44]. We compared the expressions of E2F3 and FOXM1 across the BRCA cohort (n = 1097), the BC subtypes [Luminal (n = 566), HER2+ (n = 37), triple-negative breast cancer (TNBC) (n = 116)], and the BC stages [Stage I (n = 183, stage II (n = 615), stage III (n = 247), and stage IV (n = 20)], with each other and with normal adjacent tissue (n = 114) Additionally, we compared the expression levels of the two genes in the BRCA cohort based on the TP53 mutation status. We also performed promoter region methylation analyses on tumor tissue (n = 793) and normal tissue (n = 97) using the UALCAN web server and examined the expression profiles of other input genes in BC, including E2F3 and FOXM1.

#### 4.8.2. The Survival Analysis of E2F3 and FOXM1

The Kaplan–Meier plotter (KM Plotter), an online tool designed to assess the effect of a gene on survival in various cancers using clinical data, enables researchers to conduct meta-analyses of the gene expression data to identify potential prognostic biomarkers. Kaplan–Meier survival plots can be generated to visualize the relationship between gene expression levels and patient survival outcomes. The plots offer a comprehensive analysis by integrating data from several high-quality sources, such as the Gene Expression Omnibus (GEO), the European Genome-phenome Archive (EGA), and the TCGA, and combining clinical data with gene expression profiles [45].

#### 4.8.3. The Relationship Between MiRNA vs. BRCA and MiRNA vs. RNA

TargetScan 8.0 (https://www.targetscan.org/vert_80/, accessed on 7 December 2025) was used to identify and predict the target genes of differentially expressed miRNAs [46]. The ENCORI Pan-Cancer Analysis Platform (https://rnasysu.com/encori/index.php, accessed on 7 December 2025) is developed to decode Pan-Cancer Networks involving lncRNAs, miRNAs, pseudogenes, snoRNAs, RNA-binding proteins (RBPs), and all protein-coding genes. It achieves this by analyzing their expression profiles across 32 cancer types, utilizing data from approximately 10,000 RNA-seq and 9900 miRNA-seq samples integrated from the TCGA Project [47].

#### 4.8.4. Gene-Gene Interaction Analysis

GeneMANIA is a powerful bioinformatics tool designed to identify genes that are functionally related to a given set of input genes. It leverages an extensive collection of functional association data to establish these connections. This association data encompasses various biological relationships, including protein–protein interactions, genetic interactions, shared biological pathways, gene co-expression patterns, cellular co-localization, and similarities in protein domains. By analyzing these diverse data types, GeneMANIA can predict gene function, uncover potential gene networks, and provide insights into biological processes. The protein and genetic interactions reveal how genes and their products influence one another, while the pathway data highlight the genes involved in the same biological functions. Moreover, the co-expression data identify the genes expressed simultaneously under similar conditions, suggesting a functional link. Further, the co-localization information indicates the genes whose protein products are found in the same cellular regions, and protein domain similarity suggests shared structural or functional characteristics. Overall, GeneMANIA is a comprehensive resource for researchers to explore gene relationships, predict gene function, and understand complex genetic networks within various biological systems [48].

### 4.9. Statistical Analysis

Statistical analyses were performed using the Bio-gazelle qbase PLUS 2.0 software. The quantitative real-time PCR (qRT-PCR) data were analyzed using the 2^−ΔΔCt^ method for relative quantification after normalization to the selected housekeeping gene. All the experiments were performed with at least three independent biological replicates, and each measurement was carried out in technical duplicates. The normality of the data distribution was evaluated using the Shapiro–Wilk test. For comparisons between the two groups, Student’s *t*-test was applied, while multiple group comparisons were performed using one-way analysis of variance (ANOVA) when appropriate. The data are presented as mean ± standard deviation (SD). A *p* value ≤ 0.05 was considered statistically significant.

## 5. Conclusions

This study provides strong experimental and bioinformatic evidence that miR-548c-3p exhibits a marked time-dependent downregulation in MCF-7 breast cancer cells, which is inversely associated with the progressive upregulation of the oncogenic transcription factors E2F3 and FOXM1. The most prominent inverse expression pattern was observed at the late time points, particularly at 48 h and 72 h, thus supporting a time-dependent post-transcriptional derepression mechanism. The highest expression levels of E2F3 and FOXM1 were detected in TNBC within the TCGA data; however, these observations reflect clinical datasets rather than our in vitro model, and therefore cannot be generalized across breast cancer subtypes. Moreover, the elevated expression of both genes was strongly associated with poor overall and relapse-free survival, highlighting their clinical and prognostic relevance. Collectively, these findings demonstrate that the miR-548c-3p–E2F3–FOXM1 regulatory axis represents a novel time-dependent post-transcriptional mechanism contributing to breast cancer progression. This axis may serve not only as a potential prognostic molecular signature but also as a promising therapeutic target, particularly for aggressive breast cancer subtypes such as TNBC.

## Figures and Tables

**Table 1:** List of miRNAs associated with breast cancer analyzed in this study. hsa-miR: Homo sapiens (human) microRNA; cel-miR: Caenorhabditis elegans microRNA used as a spike-in control; *SNORD61*, *SNORD68*, *SNORD72*, *SNORD95*, *SNORD96A*, *RNU6-2*: Small nucleolar RNAs and small nuclear RNA used as endogenous controls.

miRNA	miRNA	miRNA
hsa-miR-18a-5p	hsa-miR-25-3p	hsa-miR-152
hsa-miR-193b-3p	hsa-miR-26a-5p	hsa-miR-155-5p
hsa-miR-195-5p	hsa-miR-26b-5p	hsa-miR-15a-5p
hsa-miR-199a-3p	hsa-miR-27a-3p	hsa-miR-15b-5p
hsa-miR-199a-5p	hsa-miR-27b-3p	hsa-miR-16-5p
hsa-miR-19a-3p	hsa-miR-29a-3p	hsa-miR-17-5p
hsa-miR-19b-3p	hsa-miR-29b-3p	hsa-miR-181a-5p
hsa-miR-200a-3p	hsa-miR-29c-3p	hsa-miR-181b-5p
hsa-miR-200b-3p	hsa-miR-31-5p	hsa-miR-181c-5p
hsa-miR-200c-3p	hsa-miR-328	hsa-miR-181d
hsa-miR-202-3p	hsa-miR-340-5p	hsa-miR-182-5p
hsa-miR-203a	hsa-miR-424-5p	hsa-miR-186-5p
hsa-let-7a-5p	hsa-miR-132-3p	hsa-miR-10b-5p
hsa-let-7b-5p	hsa-miR-129-5p	hsa-miR-125b-5p
hsa-let-7c	hsa-miR-130a-3p	hsa-miR-125b-1-3p
hsa-let-7d-5p	hsa-miR-130b-3p	hsa-miR-128
hsa-miR-429	hsa-miR-140-5p	hsa-miR-204-5p
hsa-miR-485-5p	hsa-miR-141-3p	hsa-miR-206
hsa-miR-489	hsa-miR-145-5p	hsa-miR-20a-5p
hsa-miR-495-3p	hsa-miR-148a-3p	hsa-miR-20b-5p
hsa-miR-497-5p	hsa-miR-1	cel-miR-39-3p
hsa-miR-548c-3p	hsa-miR-100-5p	cel-miR-39-3p
hsa-miR-607	hsa-miR-107	SNORD61
hsa-miR-613	hsa-miR-10a-5p	SNORD68
hsa-miR-7-5p	hsa-miR-21-5p	SNORD72
hsa-miR-93-5p	hsa-miR-210	SNORD95
hsa-miR-96-5p	hsa-miR-212-3p	SNORD96A
hsa-miR-98-5p	hsa-miR-214-3p	RNU6-2
hsa-let-7e-5p	hsa-miR-22-3p	
hsa-let-7f-5p	hsa-miR-222-3p	
hsa-let-7g-5p	hsa-miR-223-3p	
hsa-let-7i-5p	hsa-miR-205-5p	

**Table 2:** Primer sequences for *E2F3* and *FOXM1* genes for qRT-PCR analysis in MCF-7 cells. Forward and reverse primers were designed for target genes *E2F3* and *FOXM1*, and housekeeping gene *GAPDH* was used as an internal control. All sequences are presented in 5′ to 3′ orientation.

Primer	Forward	Reverse
*E2F3*	GTCATCAGTACCTCTCAGATGG	GCAGACCAAGAGACGTATCATA
*FOXM1*	GAAGAACTCCACCCGCCACAACC	TGCTGCTGCTTAAACACCTGGTC
*GAPDH*	CAACGAATTTGGCTACAGCA	AAACTGTGAAGAGGGGCAGA

## Data Availability

All data generated or analyzed during this study are included in this published article. The bioinformatic datasets used for validation were obtained from publicly available databases, including The Cancer Genome Atlas (TCGA) via the UALCAN platform (https://ualcan.path.uab.edu), ENCORI (https://rnasysu.com/encori), and KM Plotter (https://kmplot.com).

## Abbreviations

The following abbreviations are used in this manuscript:

ER	Estrogen receptor
PR	Progesterone receptor
HER2	Human epidermal growth factor 2-receptor
miRNA	microRNA
E2F1	E2F transcription factor 1
FOXM1	Forkhead box M1
EMT	Epithelial–mesenchymal transition
TNBC	Triple-negative breast cancer
MCF-7	Breast cancer-derived cell line
TCGA	The Cancer Genome Atlas
KM Plotter	Kaplan–Meier Plotter
GEO	Gene Expression Omnibus
EGA	European Genome–Phenome Archive
Ct	Normalized threshold cycle
ECM	Extracellular Matrix
